# 1430. Eravacycline associated hypofibrinogenemia during treatment of M.abscessus

**DOI:** 10.1093/ofid/ofac492.1259

**Published:** 2022-12-15

**Authors:** Ethan Rausch, David van Duin, Luther A Bartelt, Lindsay M Daniels

**Affiliations:** UNC Medical Center, Chapel Hill, North Carolina; University of North Carolina at Chapel Hill, Chapel Hill, North Carolina; University of North Carolina School of Medicine, Chapel Hill, North Carolina; UNC Medical Center, Chapel Hill, North Carolina

## Abstract

**Background:**

Eravacycline, a novel synthetic fluorocycline, is structurally similar to tigecycline. Cases of tigecycline associated hyperfibrinogenemia have been reported in the literature, however the mechanism is not currently well described. At this time it is unknown if this is a class effect.

**Methods:**

Two cases of patients on eravacycline for treatment of *Mycobacterium abscessus* (*M.abscessus*) who received regular fibrinogen monitoring are described.

**Results:**

Patient 1 received a kidney transplant (2010) and was admitted for acute hypoxic respiratory failure secondary to COVID-19. Their course was complicated by multiple infections including disseminated *M.abscessus* with positive cultures from the lung and blood. Eravacycline 1 mg/kg BID (80 mg) was started on D1 and continued through D24. Fibrinogen levels on D1 was 448 mg/dl and on D23 were 120 mg/dl. Eravacycline was stopped and fibrinogen returned to normal range (228 mg/dl) in 5 days. Eravacycline was re-trialed at 80 mg BID and fibrinogen level on D1 was 310 mg/dl and 147 mg/dl on D8. No repeat fibrinogen levels were obtained. Patient 2 was a lung transplant recipient (2019) admitted for treatment of *M.abscessus* skin and soft tissue infection. The patient was started on eravacycline 1 mg/kg BID (90 mg) due to concerns of hypofibrinogenemia from tigecycline. On D1 of eravacycline fibrinogen was 167 mg/dl , on D19 of therapy fibrinogen was 64 mg/dl and eravacycline was stopped. Fibrinogen level returned to normal 3 days after eravacycline discontinuation (212 mg/dl).

**Conclusion:**

Similar to tigecycline, we observed eravacycline related hypofibrinogenemia. Time to onset was variable in the two cases presented. Hypofibrinogenemia was readily reversible, within 3-5 days, with drug withdrawal and reproducible in one patient with re-challenge of eravacycline. Further analysis into eravacycline related hypofibrinogenemia and its impact on coagulation outcomes are warranted based on these reports.

**Disclosures:**

**David van Duin, MD, PhD**, Achaogen: Advisor/Consultant|Allergan: Advisor/Consultant|Astellas: Advisor/Consultant|MedImmune: Advisor/Consultant|Melinta: Advisor/Consultant|Merck: Advisor/Consultant|Merck: Grant/Research Support|NeuMedicine: Advisor/Consultant|Pfizer: Advisor/Consultant|Qpex: Advisor/Consultant|Roche: Advisor/Consultant|Sanofi-Pasteur: Advisor/Consultant|Shionogi: Advisor/Consultant|Shionogi: Grant/Research Support|T2 Biosystems: Advisor/Consultant|Tetraphase: Advisor/Consultant.

